# A *Drosophila* Model to Image Phagosome Maturation 

**DOI:** 10.3390/cells2020188

**Published:** 2013-03-26

**Authors:** Tetyana Shandala, Chiaoxin Lim, Alexandra Sorvina, Douglas A. Brooks

**Affiliations:** 1 Mechanisms in Cell Biology and Diseases Research Group, School of Pharmacy and Medical Science, Sansom Institute for Health Research, University of South Australia, Adelaide, SA 5001, Australia; E-Mails: chiao_xin.lim@mymail.unisa.edu.au (C.L.); alexandra.sorvina@mymail.unisa.edu.au (A.S.); doug.brooks@unisa.edu.au (D.B.); 2 University of Adelaide, Adelaide, SA 5000, Australia

**Keywords:** *Drosophila*, *ex vivo*, *in vivo*, hemocytes, phagocytosis, *E. coli*, Rab7 GTPase, Lamp1, 14-3-3 protein

## Abstract

Phagocytosis involves the internalization of extracellular material by invagination of the plasma membrane to form intracellular vesicles called phagosomes, which have functions that include pathogen degradation. The degradative properties of phagosomes are thought to be conferred by sequential fusion with endosomes and lysosomes; however, this maturation process has not been studied *in vivo*. We employed *Drosophila* hemocytes, which are similar to mammalian professional macrophages, to establish a model of phagosome maturation. Adult *Drosophila* females, carrying transgenic Rab7-GFP endosome and Lamp1-GFP lysosome markers, were injected with *E. coli* DH5α and the hemocytes were collected at 15, 30, 45 and 60 minutes after infection. In wild-type females, *E. coli* were detected within enlarged Rab7-GFP positive phagosomes at 15 to 45 minutes after infection; and were also observed in enlarged Lamp1-GFP positive phagolysosomes at 45 minutes. Two-photon imaging of hemocytes *in vivo* confirmed this vesicle morphology, including enlargement of Rab7-GFP and Lamp1-GFP structures that often appeared to protrude from hemocytes. The interaction of endosomes and lysosomes with * E. coli* phagosomes observed in *Drosophila* hemocytes was consistent with that previously described for phagosome maturation in human *ex vivo* macrophages. We also tested our model as a tool for genetic analysis using *14-3-3ε* mutants, and demonstrated altered phagosome maturation with delayed *E. coli* internalization, trafficking and/or degradation. These findings demonstrate that *Drosophila* hemocytes provide an appropriate, genetically amenable, model for analyzing phagosome maturation *ex vivo* and *in vivo*.

## Abbreviations

IMD: immune deficiency
Rab: Ras-associated binding proteins
GFP: green fluorescent protein
Lamp1: lysosomal-associated membrane protein 1

## 1. Introduction

Phagocytosis was first defined a century ago in seminal studies by the Nobel Laureate Ilya Metchnikov [1]. Subsequently, the mechanisms for phagocytosis and the anti-microbicidal activity of phagosomes have begun to emerge [2,3]. In vertebrates, three types of cells are considered as professional phagocytes; macrophages, neutrophils, and dendritic cells [4]. Along with their crucial function in the cellular immune response, namely the degradation of microbes, they play a role in antigen presentation and adaptive immunity. *Drosophila* only exhibit innate immunity and the cellular response is carried out by hemocytes (the prototype of vertebrate macrophages). Although hemocytes do not appear to be essential for the propagation of an innate immune response and organism survival [5], they play a role in clearing exogenous objects from the hemolymph and in sensitizing humoral components of immune defense [6]. Phagocytosis involves the engulfment and internalization of, for example, bacteria into plasma membrane-derived intracellular vesicles, called phagosomes. These nascent phagosomes serve to both isolate the pathogen and to initiate other cellular processes that degrade microorganisms. The phagosome undergoes a maturation process involving sequential fusion with endosomes and lysosomes to form a fully functional phagolysosome, which has maximum degradative capacity [7]. This fusion process effectively delivers a range of lysosomal acid hydrolases to the lumen of the phagosome, which then degrades the microorganisms and other debris [8,9]. 

Small GTPases of the Rab family are involved in controlling endocytic vesicle membrane tethering and fusion, and are therefore important regulators of the phagosome maturation process [10]. For instance, Rab5, which regulates endocytosis from the plasma membrane and interaction of phagosomes with early endosomes, facilitates the recruitment of late endosomes, marked by the presence of Rab7. Rab7 is then involved in mediating the tethering of phagosomes and lysosomes. Mature phagolysosomes are characterized by the presence of the lysosome-associated membrane protein 1, or Lamp1. Lamp1 is thought to act as a structural component of lysosomes, but is also specifically required for maintaining normal microtubule activity during phagolysosome fusion events [11]. Rab GTPases are also involved in recruiting adapter proteins, such as the lysosomal trafficking regulator Lyst and hepatocyte growth factor-regulated tyrosine kinase substrate Hrs. The recruitment of these two cytoplasmic proteins to membranes can then activate the assembly of SNARE (soluble-N-ethylmaleimidesensitive-factor accessory-protein (SNAP) receptor) complexes. SNAREs control multiple membrane fusion and fission events, including the initial formation of phagosomes and subsequently phagolysosomes. SNARE proteins are found in all eukaryotic cells, playing a pivotal role in vesicular traffic within cells of the immune system, and therefore regulating the immune response [12]. 

Much of the previous knowledge regarding the process of phagosome maturation comes from cell culture and *ex vivo* mammalian cellular models [13]. There is, however, a serious gap in our knowledge as to how this process occurs in real time *in vivo*. Several studies have determined the validity of *Drosophila* embryonic hemocyte S2 and mbn(1) cells [14,15,16], and a combination of *in vivo/ex vivo* hemocytes to study phagocytosis [17]. The functional similarity of hemocytes with mammalian macrophages/neutrophils and the lack of an adaptive immune response, makes *Drosophila* an ideal model to elucidate the genetic controls for phagosome maturation [18].

## 2. Experimental Section

*Fly stocks*. Fly stocks were obtained from the Bloomington Drosophila Stock Center (Indiana University, IN, USA). For targeted expression of genes of interest, the yeast *GAL4-UAS* system was used [19]. For the phagosome maturation analysis, hemocyte-specific expression of transgenes from the *UAS* was driven by *CG-GAL4* driver [20]. All stocks used were back crossed into w^1118 ^genetic background. The protein null *14-3-3ε* mutants are semi-lethal and 40% survive to adulthood; and were used for the analysis of the *14-3-3ε* role in phagosome maturation. 

*Bacterial injection and collection of hemocytes*. One or two day old female adult flies, carrying transgenic markers of late endosomes (*CG-GAL4>UAS-Rab7-GFP*; *CG-GAL4>UAS-Lamp1-GFP*), were injected either with a fine needle dipped in a concentrated suspension of either heat-inactivated FITC-*E. coli* (Vybrant® Phagocytosis Assay Kit, Invitrogen, USA) or live *E. coli* (DH5α strain), or for controls a sterile needle. After injections, flies were kept at 25^o^C for 15, 30, 45 and 60 minutes, and then dissected to collect hemocytes in a drop of 1xPBS on poly-L-lysine or poly-L-ornithine (Sigma, USA) coated coverslip. The hemocytes were fixed and immune-stained according to a previously described protocol [21]. *E. coli* was detected with rabbit anti-*E. coli* serum (130 μg/mL; Dako, Glostrup, Denmark). Alexa Fluor® 568 Phalloidin (Invitrogen, USA) was used for detecting actin filaments. Our preliminary analysis established that one to two day old female flies could yield sufficient numbers of hemocytes in hemolymph bleeds for the analysis. This number rapidly declined with the age of the flies, presumably due to the failure to immobilize sessile hemocytes. The normal distribution and morphology of late endosomes and lysosomes was assessed in the hemocytes from non-infected siblings. At least 10 independent flies were analyzed in each experiment. Fixed and stained cells were viewed with either Zeiss LSM710 META NLO inverted microscope (Zeiss, Germany) or confocal microscope equipped with a BioRad MRC1000 scan-head and a krypton/argon laser. 

*Two-photon microscopy and in vivo imaging of Drosophila adult hemocytes. Drosophila* adult females were anesthetized for 10 minutes with Halothane (Sigma, USA) to immobilize the skeletal and smooth muscles. For *in vivo* imaging, the intact larvae *Drosophila* were coupled to the coverslip with a Carbomer 940 (Snowdrift farm, Tucson, USA) based optical coupling gel [22]. A Zeiss LSM710 META NLO inverted microscope (Zeiss, Germany) was supplemented with a two-photon Mai-Tai^®^, tunable Ti:Sapphire femtosecond pulse laser (710-920 nm, Spectra-Physics, USA). Endogenous fluorescence was detected using the following setting (two-photon excitation wavelength 830 nm, main beam splitter MBS 690+, emission interval 371-436 nm). GFP-fluorescence was detected using the following setting (two-photon excitation wavelength 830 nm, beam splitter MBS 690+, emission interval 475 nm-552 nm). For Z-axis imaging, optical sections were acquired with 1 μm intervals. The images through the adult cuticles (in areas with minimal hair/bristle numbers) were acquired using a Plan-APOCHROMAT 63X/ NA1.4 oil immersion objective. 

*Image processing.* The final preparation of the images was conducted with Adobe Photoshop CS6 (Adobe Systems Inc, USA). Confocal micrographs in Figure 2, Figure 4 and Figure 6 represent seven 0.5 µm optical sections that were merged using Photoshop CS6 (Adobe, USA). The movies present three dimensional reconstructions using the optical sections, which were processed using Volocity 3D Image Analysis software (PerkinElmer, USA), and were used to validate the bacterial ingestion by hemocytes. The movies illustrating three dimensional reconstruction of *in vivo* Z-stacks were assembled using Zen 2011 software (Zeiss, Germany).

## 3. Results and Discussion

### 3.1. Establishing a Model of Phagosome Maturation

**Figure 1:**
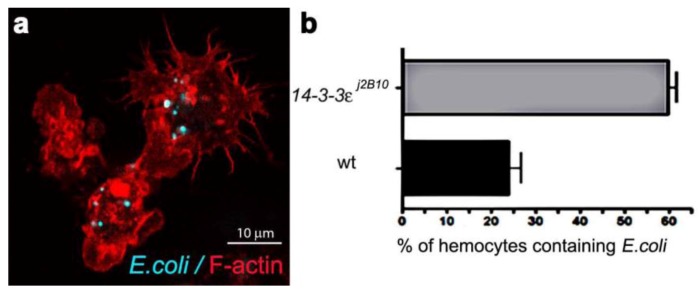
**(a)** Confocal micrograph showing hemocytes (stained with Phalloidin-Alexa 568 for actin-filaments, red) containing FITC-labeled *E. coli* (turquoise punctae). **(b) **Enumeration of wild-type and *14-3-3ε* protein null mutant hemocytes containing *E. coli*, one hour after infection.

**Figure 2:**
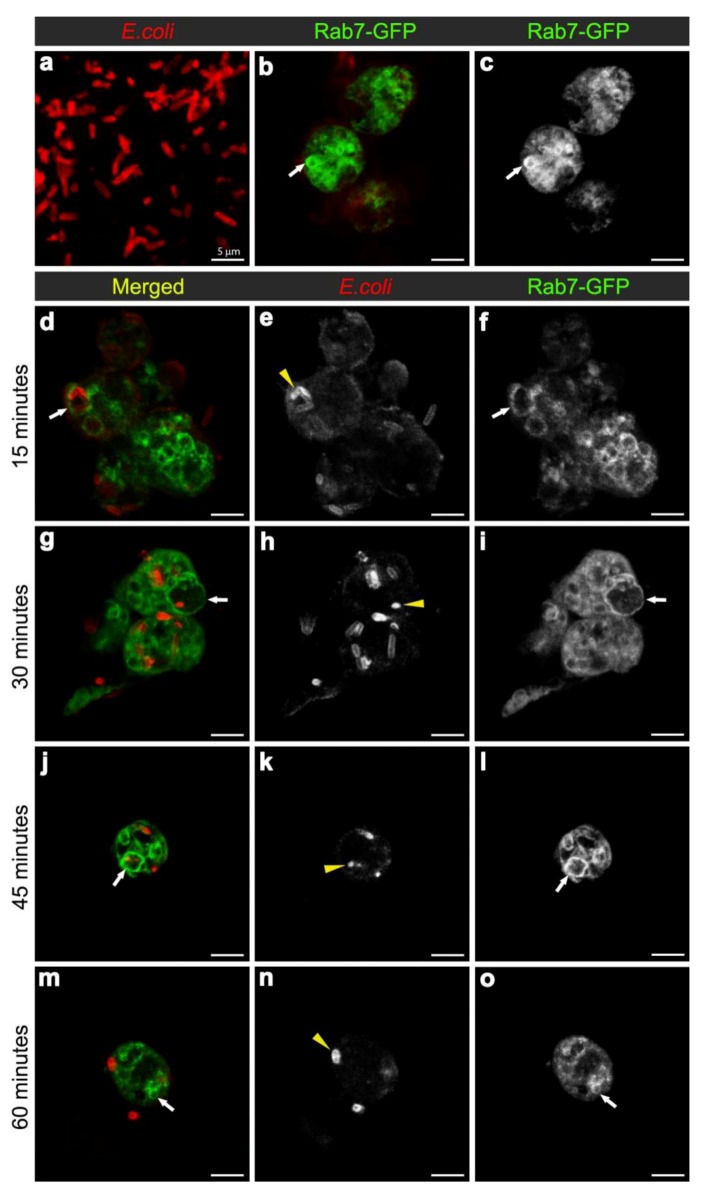
Confocal micrographs of intact *E. coli* (red in **(a)**, for scaling purposes to illustrate the size and rod-shape of free *E. coli*), and Rab7-GFP positive vesicles (expressed using *CG-GAL4>Rab7-GFP*): in uninfected hemocytes with vesicles in green **(b)** and grayscale **(c)** and depicted by arrows; and in hemocytes at 15 **(d-f)**, 30 **(g-i)**, 45 **(j-l)**, and 60 **(m-o)** minutes after infection with *E. coli* (red in **d**, **g**, **j, m**; and grayscale in **e**, **h**, **k, n,** also depicted by yellow arrowheads). Rab7-GFP positive vesicles (green in **d**, **g**, **j**, **m**; and grayscale in **f**, **i**, **l, o**) are depicted with arrows. Scale bars = 5 µm as labeled in **(a)**.

Phagosome maturation involves the successive interaction of phagosomes with endosomes and lysosomes to form acidic phagolysosomes, which provide a degradative environment for pathogen removal. Although cell based genetic screens have been carried out to define some key members of the molecular machinery required for phagocytosis [23,24,25,26], the process of phagosome maturation has not been studied *in vivo,* hence the establishment of the *Drosophila* model herein. Firstly, we determined the efficiency of phagocytosis, by injecting one to two day old w^1118 ^wild-type females, with a needle coated in either heat-inactivated *E. coli* (labeled with FITC) or a control sterile needle (Figure 1a). The phagocytic index was calculated from the percentage of hemocytes with internalised *E. coli*. One hour post infection, 24% of hemocytes (n = 163), detected by Phalloidin-Alexa 568 staining of peri-membranous filamentous actin, contained FITC-positive bacterial particles (Figure 1b), providing a phagocytic index sufficient for the analysis of phagosome maturation. Transgenic flies carrying the endosome-lysosome markers *Rab7-GFP* and *Lamp1-GFP* were used for the phagosome maturation analysis. Adult females were injected either with a needle coated in unstained live *E. coli* (to avoid GFP and FITC spectral overlap) or control bacterial culture medium, LB-broth. To investigate a time course for phagosome maturation, hemocytes were harvested onto coverslips at 15, 30, 45 and 60 minutes post infection and immediately fixed. The *E. coli* were detected by immune-staining with rabbit-anti-*E. coli* and anti-rabbit-Cy3 antibodies (Figure 2a, Figure 4a; depicting intact bacteria), and their distribution analyzed in relation to GFP-labeled endosome-lysosome compartments (Figure 2, Figure 4).

### 3.2. E. coli was Localised to Rab7-Positive Phagosomes at 15-45 Minutes after Infection

**Figure 3:**
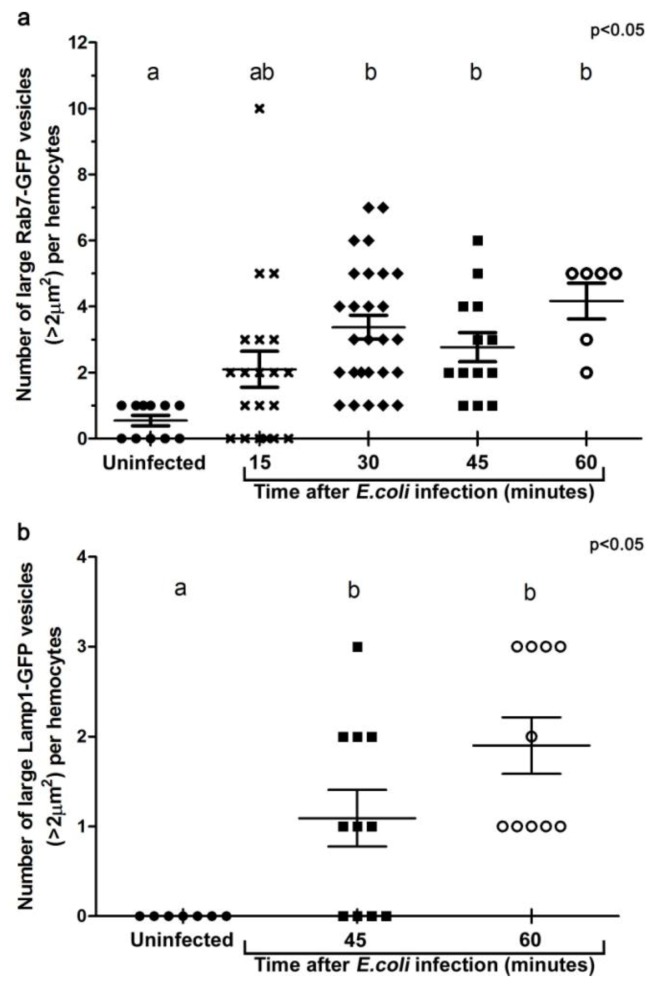
Histograms illustrating the changes in the number of large Rab7-GFP **(a)**, and Lamp1-GFP **(b)** compartments per hemocyte when examined *ex vivo* at designated time points after *in vivo* infection. For each group, at least five individual adult female flies were analyzed. Each symbol represents a single hemocyte; and the respective number of vesicles per hemocyte displayed on the Y-axis. The changes in the number of large vesicles per hemocyte is presented as mean±SEM. One-way ANOVA analysis of variance showed significant differences between group means (*P* < 0.05). Tukey’s multiple comparison test showed significant differences between the means of groups depicted by the different letters on the bars (*P* < 0.05).

To examine the maturation of bacterial phagosomes in hemocytes, we first investigated *E. coli* distribution in relation to Rab7-GFP endosome compartments. In hemocytes isolated from uninfected female flies, only small Rab7-GFP positive vesicles were observed, with some ubiquitous Rab7-GFP signal in the cytoplasm (Figure 2b-c). After *in vivo* infection, *E. coli* were detected in *ex vivo* isolated hemocytes and the relative distribution of Rab7-GFP changed, with a reduction in the cytoplasmic signal and an increase in the signal associated with Rab7-GFP positive vesicular compartments (Figure 2d,f); due to a significant increase in size from 30 min after infection (Figure 3a). The presence of bacteria within hemocyte vesicles was demonstrated by analyzing three-dimensional reconstructions from a series of confocal Z-axis sections, where the low level of cytoplasmic GFP signal outlined the hemocyte shape (Movie 1). In 25% of these *ex vivo* hemocytes (n = 20), some *E. coli* were detected in enlarged Rab7-GFP positive phagosomes as early as 15 minutes after infection, with some *E. coli* located either on the surface of hemocytes or entering the hemocytes (Figure 2d-f). At this time point, the bacteria had a characteristic rod-shape (Figure 2d,e), which was similar to that observed for control bacteria prior to injection (Figure 2a), suggesting that the former were still intact. Some Rab7-GFP phagosomes (Figure 2d,f, arrow) contained multiple intact bacteria (Figure 2e, arrowhead). 

Bacteria or fragments thereof persisted in Rab7-GFP compartments for 30-45 minutes post infection (Figure 2d-l). At 30 minutes, there was an increased number of *E. coli* observed within hemocytes, with 55.6% hemocytes (n = 27) with Rab7-GFP positive vesicles containing *E. coli* (Figure 2g-I; Movie 1). However, it was not possible to distinguish between multiple phagocytic events and replication of *E. coli* within hemocytes, the latter was unlikely given that the replication time for *E. coli* is ~60 minutes [27]. While some of the internalized bacteria appeared to be intact at 30 minutes after infection, there was also small punctate *E. coli*-positive staining, suggesting that some bacterial degradation had occurred (Figure 2h, arrowhead). Moreover, by 45 minutes post infection there appeared to be a reduction in the number of hemocytes (to 35.7%; n = 14) with Rab7-GFP positive vesicles containing *E. coli* (Figure 2j-l), when compared to the 30 minutes time point (Figure 2g-i); and most of the *E. coli*-signal was small and punctate with no obvious intact *E. coli* (Figure 2k, arrowhead). After 60 minutes post infection, little or no Rab7-GFP was associated with *E. coli* phagosomes (Figure 2m-o; Movie 2). The timing for the detection of *E. coli* in Rab7 phagosomes observed here was consistent with previous *ex vivo* findings for mammalian macrophages, where phagosomes have been reported to acquire the small GTPase Rab7 at approximately 10 to 30 minutes after infection [8]. 

### 3.3. E. coli was Localised to Lamp1-Positive Phagosomes 45 Minutes after Infection

**Figure 4:**
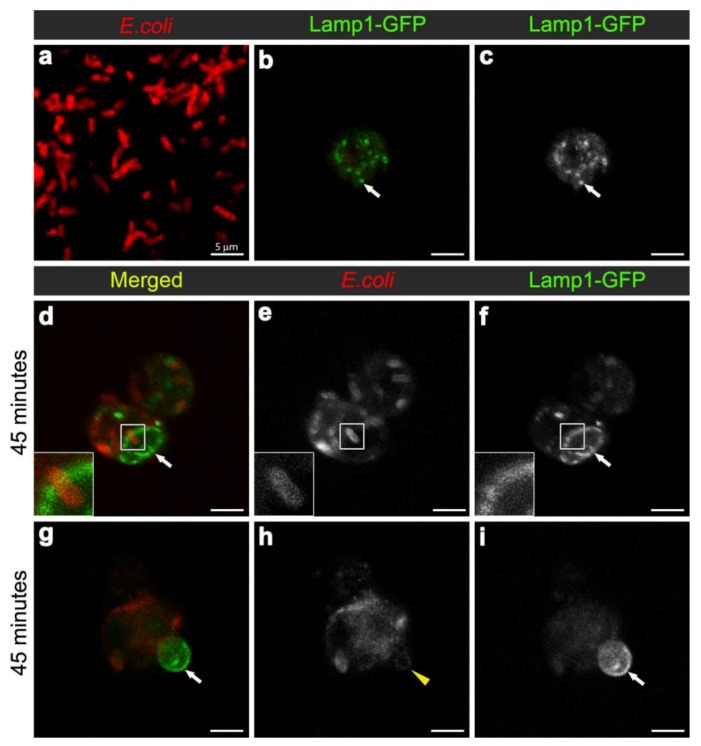
Confocal micrograph of intact *E. coli* (red in **(a)**, replicated from Figure 2a, for scaling purposes to illustrate the size and rod-shape of free *E. coli*). Confocal micrographs of Lamp1-GFP positive vesicles (expressed using *CG-GAL4>Lamp1-GFP*): in uninfected wild-type hemocytes as green **(b)** and grayscale **(c)** and depicted by arrows; and in hemocytes 45 minutes after infection as green **(d, g)** and grayscale **(f, i)** and depicted by arrows; with *E. coli* visualized as red **(d, g)**, and grayscale **(e, h)**. Inset in **(d-f) **depicts intact *E. coli* entering enlarged Lamp1-GFP compartment, framed in the main panels. Arrows in **(g, i)** depict Lamp1-GFP-positive bud in wild-type hemocyte, containing a low *E. coli-*positive signal designated by arrowhead in **(h)***.* Scale bars = 5 µm as labeled in **(a)**.

To analyse the formation of phagolysosomes, adult females carrying the late endosome/lysosome marker Lamp1-GFP, were infected with *E. coli*. In hemocytes from uninfected females, Lamp1-GFP positive vesicles were observed as small bright green punctae, distributed throughout the cytoplasm of the hemocytes (Figure 4b-c). In contrast, 45 minutes after infection, there were enlarged Lamp1-GFP vesicles, containing *E. coli*-positive signal (Figure 3b, Figure 4d,f; and reconstructed in Movie 3), which were often localised at the periphery of the hemocytes (Figure 4d-f; and 3D reconstruction in Movie 3). This was consistent with previous findings in mammalian macrophages, which showed that bacterial phagosomes preferentially fuse with lysosomes at approximately one hour after infection [8,9]. There were also very large Lamp1-GFP positive structures that apparently extended either within or from the hemocyte (Figure 4g-i). Unlike the intracellular Lamp1-GFP (Figure 4d-f) and Rab7-GFP positive compartments (Figure 2d-l), these protruding Lamp1-GFP structures appeared to contain no intact *E. coli* and limited *E. coli* specific signal (Figure 4g-i). However, we could not exclude the possibility that the antibody had a limited capacity to detect small bacterial fragments, which may have resulted from phagolysosomal degradation in these compartments. Indeed, Lamp1-positive lysosomes were first discovered in mammalian hematopoietic cells, such as macrophages, natural killer cells, and neutrophils, and are known to contain hydrolytic enzymes that can be deployed to degrade microorganisms [28,29].

**Figure 5:**
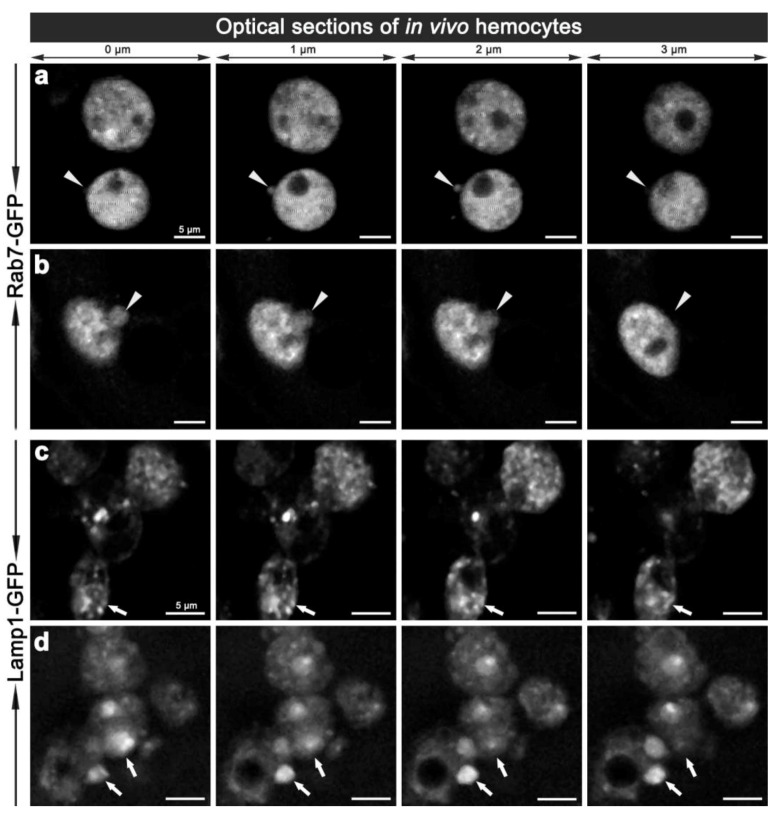
Two-photon micrographs illustrating the *in* vivo morphology of adult hemocytes carrying *CG-GAL4>Rab7-GFP* **(a, b)** and *CG-GAL4>*Lamp1-GFP **(c, d)** markers. Each row shows consecutive optical 1 μm thin sections along the Z-axis with 0.5 μm steps in-between, which are representative of Movie 4 and 4a **(a)**, and Movie 5 **(b)**. Arrowheads **(a, b)** depict the vesicles protruding from the hemocyte surface. Arrows **(c, d)** depict small or enlarged Lamp1-GFP compartments. Scale bars = 5 μm.

To validate the changes observed *ex vivo*, we examined the morphology of hemocyte phagosomes *in vivo*, employing two-photon fluorescence microscopy, which offers significant advantages over single-photon confocal fluorescence imaging: with increased depth of penetration; reduced tissue damage from the higher wavelength light; decreased photo-bleaching; and less auto-fluorescence; thereby providing better Z-axis penetration and specificity for live tissue imaging. Imaging of live hemocytes within adult females also showed enlarged Rab7-GFP and Lamp1-GFP compartments (Movie 4a, 7a, 8a), which again were often observed protruding from the hemocytes (Figure 5; Movie 4,5,6,8). This dynamics of hemocyte membranes appears to be intrinsic, and was observed in some uninfected hemocytes. This is analogous to vesicles that have been reported to bud off the plasma membrane of neutrophils and tumor cells; resulting in the release of different membrane vesicles, including exosomes, that vary between 50–1,000 nm in diameter and have purported roles in immune responses [30,31,32]. Interestingly, we also observed ~100 nm diameter extracellular vesicles freely floating in the hemolymph of live adults (Movies 4,6,7). These putative exosome related vesicles, have been underappreciated in *Drosophila*, but mammalian exosomes are known to be play a pivotal role in many intercellular signaling events, e.g., involved in organism development and immunity [33,34]. It would therefore be interesting to further investigate the biology of these enlarged Rab7-GFP and Lamp1-GFP positive compartments and exosomes to address the question of whether they contain bacterial fragments, cytokines and or antimicrobial peptides, and whether the depletion of these compartments might interfere with immune communication during an infection in *Drosophila*. This analysis would be enriched by monitoring the real-time changes in the morphology of these vesicles as well as their motility and fate *in vivo*.

### 3.4. Towards Dissecting the Genetic Control of Phagosome Maturation

**Figure 6:**
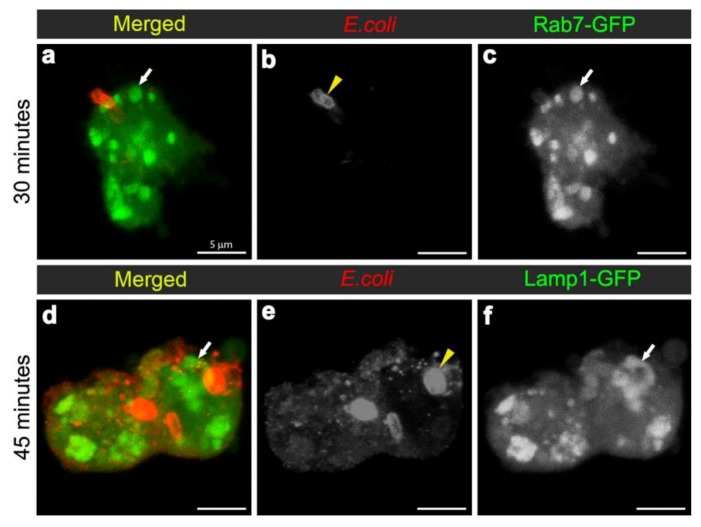
Confocal micrographs depicting hemocytes isolated from the *14-3-3ε* protein null mutant female flies at 30 minutes **(a-c)** and 45 minutes **(d-f)** after infection with *E. coli* (red in **a, d** and grayscale in **b, e**). Confocal micrographs also show Rab7-GFP-positive vesicles (green in **a**; grayscale in **c**) and Lamp1-GFP positive vesicles (green in **d**; and grayscale in **f**) and depicted by arrows. Scale bars = 5µm as labeled in **(a)**.

One of the major aims of *in vivo* modeling is dissecting the genetic pathway of phagosome maturation in its natural environment. We opted to analyze *14-3-3ε* protein null mutants, as our previous study demonstrated that *14-3-3ε* mutant larvae had impaired innate immunity, with a specific defect in the secretion of anti-microbial peptides [21]. Moreover, a potential role for *14-3-3ε* and *14-3-3ζ* genes in regulating phagocytosis was predicted by an RNAi screen in Drosophila S2 embryonic hemocyte cell line; [23,35,36]. Here, there was an abnormal accumulation of phagocytosed FITC-labeled *E. coli* in *14-3-3ε* mutant hemocytes when compared to the wild-type hemocytes (one hour post infection, Figure 1). This could either suggest that *14-3-3ε* mutant hemocytes have increased capacity for phagocytosis, or considering the time point post infection, more likely a defect in phagolysosome maturation/degradative capacity. We therefore used our new model to explore phagosome maturation upon the loss of 14-3-3ε protein. Thirty minutes after *in vivo* infection with *E. coli,* hemocytes isolated from *14-3-3ε* mutant adults had large Rab7-GFP positive vesicles in the cytoplasm (Figure 6a,c), which, however, rarely contained *E. coli*; in contrast to wild-type control hemocytes, which had large Rab7-GFP positive phagosomes often containing multiple bacteria (Figure 2g,i). Moreover, there were very few bacteria within the mutant hemocytes, and some were still located at the surface of *14-3-3ε* mutant hemocytes (Figure 6a,b). The small number of *E. coli* observed in *14-3-3ε* mutants hemocytes at this time-point was consistent with delayed phagocytosis, which has been reported following the depletion of the other *Drosophila* homologue, 14-3-3ζ; which is not surprising, given that these two proteins function as heterodimers [36]. 

In contrast to wild-type controls (Figure 4d-f) at 45 minutes after infection with *E. coli*, *14-3-3ε* mutant hemocytes contained some *E. coli*-positive signal, which, however, showed little to no overlap with Lamp1-GFP vesicles (Figure 6d-f). Together, this suggested that the loss of 14-3-3ε protein might disrupt bacterial internalization as well as bacterial delivery into Rab7 and then Lamp1-positive phagolysosome compartments, consequently reducing the efficacy of bacterial degradation. We concluded that the phagosome formation and maturation were most likely disrupted in 14-3-3ε protein null mutants.

## 4. Conclusions

*Drosophila melanogaster* provides a valuable model to study an innate immune response because it does not possess adaptive immunity that might complicate the analysis. Whilst much has been learned about eukaryote humoral immune responses, the cellular innate immune response is still to be unraveled [37]. In particular, phagosome maturation has not been previously investigated *in vivo*, either in *Drosophila* or in mammals [38]. This is partly because mammalian phagocytes are not easily accessed *in vivo* [39]. By taking advantage of this classic genetic model, and combining *in vivo* infection/phagosome maturation and both *ex vivo* and *in vivo* analysis approaches*,* we showed that the process of phagosome maturation in *Drosophila* hemocytes resembles that of *ex vivo* and *in vitro* vertebrate models. Moreover, employing transgenic Rab7-GFP and Lamp1-GFP markers allowed us to detect the novel morphological features of phagosome maturation in *Drosophila* hemocytes. This suggests that hemocytes could serve as a valid model for studying the process of phagosome maturation *in vivo*, and this could be used to assess effects of various developmental and physiological environments. For instance, by comparing the phagosome maturation in hemocytes from larva, from young adults (as in this study), and from mature adults, it might be possible to estimate the contributing effect of tissue remodeling (occurring in late larvae and young adults) on the phagocytic potential of hemocytes. In addition, using the natural infection route rather than injection, whereby the barrier function of the cuticles is compromised, would eliminate any possible contribution from the wounding response. Further research is also required to fully characterize phagosome maturation in hemocytes exposed to other, more pathogenic bacterial strains for *Drosophila*, as described in [17]; and employing other markers such as Rab5 (early endosomes) and Rab11 (recycling endosomes), SNARE (fusion machinery) and Lyst and Hrs (adaptor proteins). The genetic depletion of this machinery will provide further insight into the sequential mechanism of phagosome maturation and its regulation. In this regard, our non-invasive two-photon imaging of the adult hemocytes *in vivo* potentially provides a model system with which to monitor the process of phagosome maturation, giving information on the cellular innate immune response in real time and space.

## Electronic Supplementary Information (ESI)

**Movie 1.** Three-dimensional reconstruction of the confocal optical Z-axis sections of the Rab7-GFP hemocyte containing *E. coli* 30 minutes post infection (see Figure 2g).

**Movie 2.** Three-dimensional reconstruction of the confocal optical Z-axis sections of the Rab7-GFP hemocyte containing *E. coli* 60 minutes post infection (see Figure 2m).

**Movie 3.** Three-dimensional reconstruction of the confocal optical Z-axis sections of the Lamp1-GFP hemocyte containing *E. coli* 45 minutes post infection (see Figure 4d).

**Movie 4.** Three-dimensional reconstruction of the two-photon optical Z-axis sections of the Rab7-GFP *in vivo* hemocyte from uninfected female.

**Movie 4a.** The stacks of two-photon optical Z-axis sections of the Rab7-GFP *in vivo* hemocyte from uninfected female (as in Movie 4), to highlight the intracellular vesicles.

**Movie 5**. Three-dimensional reconstruction of the two-photon optical Z-axis sections of the Rab7-GFP *in vivo* hemocyte 30 minutes post infection.

**Movie 6.** Three-dimensional reconstruction of the two-photon optical Z-axis sections of the Rab7-GFP *in vivo* hemocyte 30 minutes post infection.

**Movie 7.** Three-dimensional reconstruction of the two-photon optical Z-axis sections of the Lamp1-GFP *in vivo* hemocyte 30 minutes post infection.

**Movie 7a.** The stacks of two-photon optical Z-axis sections of the Lamp1-GFP *in vivo* hemocyte (as in Movie 7), to highlight the intracellular vesicles.

**Movie 8.** Three-dimensional reconstruction of the two-photon optical Z-axis sections of the Lamp1-GFP *in vivo* hemocyte 30 minutes post infection.

**Movie 8a.** The stacks of two-photon optical Z-axis sections of the Lamp1-GFP *in vivo* hemocyte (as in Movie 8), to highlight the intracellular vesicles.

Supplementary File 1 Supplementary Materials (RAR, 20996 KB)
